# Decomposing socioeconomic inequality in poor mental health among Iranian adult population: results from the PERSIAN cohort study

**DOI:** 10.1186/s12888-020-02596-y

**Published:** 2020-05-13

**Authors:** Farid Najafi, Yahya Pasdar, Behzad Karami Matin, Satar Rezaei, Ali Kazemi Karyani, Shahin Soltani, Moslem Soofi, Shahab Rezaeian, Alireza Zangeneh, Mehdi Moradinazar, Behrooz Hamzeh, Zahra Jorjoran Shushtari, Mansour sajjadipour, Saeid Eslami, Maryam khosrojerdi, Sahar Shabestari, Amir Houshang Mehrparvar, Zahra Kashi, Azim Nejatizadeh, Mohammadreza Naghipour, Shahrokh Sadeghi Boogar, Ali Fakhari, Bahman Cheraghian, Haydeh Heidari, Parviz Molavi, Mohammad Hajizadeh, Yahya Salimi

**Affiliations:** 1 Research Center for Environmental Determinants of Health, Health Institute, Kermanshah University of Medical Sciences, Kermanshah, Iran; 2 Nutritional Sciences Department, School of Public Health, Kermanshah University of Medical Sciences, Kermanshah, Iran; 3 Social Development and Health Promotion Research Center, Health Institute, Kermanshah University of Medical Sciences, Kermanshah, Iran; 4 Social Determinants of Health Research Center, University of Social Welfare and Rehabilitation Sciences, Tehran, Iran; 5 South Tehran Health Center, Tehran University of Medical Sciences, Tehran, Iran; 6 Pharmaceutical Research Center, Pharmaceutical Research Institute, Mashhad University of Medical Sciences, Mashhad, Iran; 7 Cellular and molecular research center, school of medicine, Sabzevar university of medical sciences, Sabzevar, Iran; 8 Health Promotion Research Center, Zahedan University of Medical Sciences, Zahedan, Iran; 9 Industrial Diseases Research Center, Shahid Sadoughi University of Medical Sciences, Yazd, Iran; 10 Diabetes Research Center, Mazandaran University of Medical Sciences, Sari, Iran; 11 Molecular Medicine Research Center, Hormozgan University of Medical Sciences, Bandar Abbas, Iran; 12 Gastrointestinal and liver disease research center, Guilan University of medical sciences, Rasht, Iran; 13 Gastroenterohepatology Research Center, Shiraz university of medical sciences, Shiraz, Iran; 14 Research Center of psychiatry and behavioral sciences, Tabriz University of Medical sciences, Tabriz, Iran; 15 Department of Biostatistics and Epidemiology, school of public health, Ahvaz jundishapur university of medical sciences, Ahvaz, Iran; 16 Modeling in Health Research Center, Shahrekord University of Medical Sciences, Shahrekord, Iran; 17 Digestive Disease Research Center, Ardabil University of Medical Sciences, Ardabil, Iran; 18 School of Health Administration, Faculty of Health, Dalhousie University, Halifax, Canada; 19 Social Development and Health Promotion Research Center, Health Institute, Kermanshah University of Medical Sciences, Kermanshah, Iran

**Keywords:** Mental health, Socioeconomic inequality, Concentration index, Decomposition

## Abstract

**Background:**

Socioeconomic inequality in mental health in Iran is poorly understood. This study aimed to assess socioeconomic inequality in poor mental health among Iranian adults.

**Methods:**

The study used the baseline data of PERSIAN cohort study including 131,813 participants from 17 geographically distinct areas of Iran. The Erreygers Concentration index (E) was used to quantify the socioeconomic inequalities in poor mental health. Moreover, we decomposed the *E* to identify factors contributing to the observed socioeconomic inequality in poor mental health in Iran.

**Results:**

The estimated E for poor mental health was − 0.012 (95% CI: − 0.0144, − 0.0089), indicating slightly higher concentration of mental health problem among socioeconomically disadvantaged adults in Iran. Socioeconomic inequality in poor mental health was mainly explained by gender (19.93%) and age (12.70%). Region, SES itself, and physical activity were other important factors that contributed to the concentration of poor mental health among adults with low socioeconomic status.

**Conclusion:**

There exists nearly equitable distribution in poor mental health among Iranian adults, but with important variations by gender, SES, and geography. These results suggested that interventional programs in Iran should focus on should focus more on socioeconomically disadvantaged people as a whole, with particular attention to the needs of women and those living in more socially disadvantaged regions.

## Background

Mental health disorders are one of the global leading causes of morbidity and mortality influence on several aspects of life including quality of life, physical well-being, social cohesion, and productivity [1]. One systematic review by Steel et al. reported that the global prevalence of most common mental health disorders including anxiety and substance use disorders ranged between 16 and 19% [2]. Based on the 2017 Global Burden of Disease report, mental health disorders are responsible for 14% of age-standardized years lived with disabilities in the last three decades [3]. The prevalence of the mental health disorders in Iran has been shown an increase from 21% in 1999 to 24% in 2011 [4].

It is increasingly known that poor mental health has been disproportionately distributed across socioeconomic groups in a population [5, 6]. Several studies indicated that the distribution of good mental health is heavily skewed towards the higher socioeconomic groups [5, 7, 8]. A systematic review of literature by Lund et al.(2010) showed that there is a convincing evidence of association between poverty and poor mental health [5].

As many structural, social, and environmental factors have attributed to the socioeconomic inequality in poor mental health, underlying mechanisms of association between SES and poor mental health seems to be complex [9]. The poor mental health can be one of the determinants and also consequences of the socioeconomic inequality [9]. However, evidence on socioeconomic inequality in mental health from low- and middle-income countries including Iran is scarce. A context-based study for enhancing the current knowledge of socioeconomic inequalities in poor mental health is greatly warranted. Given the preventable nature of the socioeconomic inequality, providing related information on the poor mental health distribution would be useful for understanding the burden of the problem, guiding policy makers and developing practical preventive interventions. Thus, the aim of present study is to quantify the extent of socioeconomic-related inequality in poor mental health among Iranian adults and to understand determinants of socioeconomic inequality in poor mental health.

## Methods

### Data

We used the baseline data of Prospective Epidemiological Research in IrAN (PERSIAN) that included the data on 131,813 Iranians aged 35 years and older, from 14 provinces of Iran, in 17 geographically distinct cohort sites. These cohort sites include Kermanshah, Guilan, Fasa, Tabriz, Kharameh, Mazandaran, Zahedan, Yazd, Rafsanjan, Ahvaz, Shahrekurd, Bandar Abbas, Uromieh, Ardabil, Sabzevar, Mashhad, Yasuj, Kavar. As data of Fasa, Kavar and Kharameh sites came from Fars province, and Sabzevar and Mashahd from Razavi Khorasan province; therefore, the data in from the same sites of these provinces were merged. Because the process of recruitment has not been completed in time of data analysis, the Yasuj cohort was excluded from this study. In each site, men and women aged 35–70 years, residing within the PERSIAN Cohort sites are invited to participate in the study. Other inclusion criteria included: being of Iranian descent and living in the designated areas for at least 9 months of the year. Anyone with physical or psychological disabilities, that unable to complete the enrollment process was excluded from the study. This yielded a total final sample size of 130,078 adults aged 35–70 years old. More details about PERSIAN Cohort study were presented elsewhere [10, 11]. The characteristics of included cohort sites are described in Appendix 1.

### Measures

Poor mental health was defined as a binary variable based on self-report of related treatments for at least 3 month during the past year which recorded by a general physician: Citalopram, Escitalopram, Sertraline, Paroxetine, Clozapine, Quetiapine, Risperidone, Haloperidol, Chlorpromazine, Olanzapine, Aripiprazole, Fluphenazine, Perphenazine, Trifluoperazine, Fluoxetine, Valproate sodium, Lamotrigine, Alprazolam, Clonazepam, Lorazepam, Flurazepam, Buspirone, Zolpidem, Lithium, Carbamazepine, Tranylcypromine, Venlafaxine, Fluvoxamine, Trazodone, Duloxetine, Oxcarbazepine, Doxepin, Maprotiline, Trimipramine, Clomipramine, Nortriptyline, Desipramine, Amitriptyline. Although, we had access to self-reported past history of depression and any other mental health problems, diagnosed by physician, we did not include such variables in our definition as we aimed to investigate about inequality in mental health problems under the treatment. Using principal component analysis, the socioeconomic status (SES) variable was constructed by assessing ownership of household assets, and educational level of individuals. Twenty six items i.e. having car, motorcycle, bicycle, refrigerator, freezer, radio, stove, vacuum machine, personal computer, CD/DVD player, sewing machine, cooler, washing mashing, microwave, central heating, having kitchen, bathroom, use of natural gas for cooking, per capita house area per capita rooms, access to piped drinking water, electricity, telephone, internet, sewage network, and educational level were used in the construction of SES indicator. The SES index was grouped into five quintiles, where the 1st quintile represents the poorest group and the 5th quintile represents the richest one. Dummy variables for the age group, marital status, body mass index (BMI), smoking status, SES quantiles, and 14 sites of PERSIAN cohort were included in the analysis as determinants of poor mental health.

### Statistical analysis

#### Measuring and decomposing poor mental health inequality

The concentration index (C) approach [12, 13] was used to measure socioeconomic inequality in poor-mental health outcome. The C is based on the Concentration curve which plots cumulative proportion of population ranked in ascending order of SES in x-axis and cumulative proportion of poor mental health in y-axis. The C is defined as twice the area between the concentration curve and line of perfect equality (i.e., 45-degree line). Formally, the C can be calculated as:

1 $$ C=\frac{2}{\mu}\mathit{\operatorname{cov}}\left({Y}_i,{R}_i\right) $$

Where *μ* is the mean of poor mental health disorder, *y*_*i*_ is the mental health disorder status of the *i* th individual and *R*_*i*_ is the fractional rank that individual *i* represents in total population ranked by SES. The C is bounded between the values of − 1 and + 1. Negative values imply that poor mental health is more concentrated among the poor people and positive values imply that poor mental health is more concentrated among rich people. If the C is equal to zero it suggests that there is no socioeconomic inequality in mental health disorder. If health outcome variable in bounded, the estimated value of the C is not between − 1 and + 1. Thus, we used Erreygers Concentration index (E) [14] to account for the bounded nature of binary health outcome variables. The formula for the E is as follows:

2 $$ E=\frac{4\mu }{y^{max}-{y}^{min}}C. $$

Where *y*^*max*^ and *y*^*max*^ are the minimum and maximum value of the bounded variable (i.e., one and zero for binary variable).

The E can be decomposed to identify the contributions of relevant factors to socioeconomic inequality in mental health disorder. Assuming a linear relationship between mental health disorder and a set of *k* explanatory variables *x*, the E can be expressed as a weighted sum of the partial Concentration index for the explanatory factors of socioeconomic inequality, *C*_*k*_ as:

3 $$ E=4\ \left[{\sum}_k\left({\beta}_k{\overline{x}}_k\right){C}_k+G{C}_{\varepsilon}\right] $$

Where ${\overline{x}}_k$ is the means of explanatory variables, *β*_*k*_ is the coefficient on explanatory variable *k* obtained from the generalized linear model of the binomial family with a logit link function linking mental health disorder to the explanatory variables, and *GC*_ε_ is the generalized C for the error term. If the value of the contribution of variable *k* is *θ*, for both positive (negative) signs, then the inequality in poor mental health would decrease (increase) by *θ* percent if the variable was to become equally distributed across the socioeconomic groups. Normal-based 95% bootstrap confidence intervals with 1000 replication were calculated. The level of significance (alpha level) in all analyses was set at 0.05. Records with missing data were excluded because the amount of missing data was small less than 1% and assumed to be missing at random. Statistical analysis procedures were conducted using STATA 11 [15] and the Es were calculated using Stata’s “conindex” command [16].

## Results

### Descriptive results

As shown in Table 1, from total of 131,813 participants, nearly 45% of the study sample were male; most participants were 35–40 years (20.83%) and belonged to the overweight category (40.76%). The majority of participants were married (90. 94%), non-smoker (86%) and non-alcohol user (90.96%). 11% of the participants were water-pipe user.

**Table 1:** Characteristics of study participants by mental health disorders (*n* = 130,078)

	All participants	Poor mental health	Good mental health
	N (%)	N (%)	N (%)
**Age groups**			
35–40	27,094 (20.83)	1094 (4.04)	26,000 (95.96)
40–45	24,195 (18.60)	1189 (4.91)	23,006 (95.09)
45–50	22,489 (17.29)	1468 (6.53)	21,021 (93.47)
50–55	20,160 (15.50)	1601 (7.94)	18,559 (92.06)
55–60	17,442 (13.41)	1431 (8.20)	16,011 (91.80)
60–65	12,154 (9.34)	984 (8.10)	11,170 (91.90)
65 >	6544 (5.03)	581 (8.88)	5963 (91.12)
**Gender**			
Male	58,251 (44.78)	2499 (4.29)	55,752 (95.71)
Female	71,827 (55.22)	5849 (8.14)	65,978 (91.86)
**Marital status**			
Single	2953 (2.27)	176 (5.96)	2777 (94.04)
Married	118,290 (90.94)	7239 (6.12)	111,051 (93.88)
Divorced	1447 (1.11)	125 (8.64)	1322 (91.36)
widowed	7305 (5.62)	802 (10.98)	6503 (89.02)
other	83 (0.06)	6 (7.23)	77 (92.77)
**Water-pipe use**			
No	114,594 (88.92)	7395 (6.45)	107,199 (93.55)
Yes	14,284 (11.08)	949 (6.47)	13,335 (93.53)
**Alcohol Use**			
No	117,225 (90.96)	7737 (6.60)	109,488 (93.40)
Yes	11,652 (9.04)	606 (5.20)	11.046 (94.80)
**Drug use**			
No	113,514 (88.08)	7143 (6.29)	106,371 (93.71)
Yes	15.363 (11.92)	1201 (7.82)	14,162 (92.18)
**Smoking status**			
Non-smoker	91,174 (77.81)	6636 (7.28)	84,538 (92.72)
Ex-smoker	9166 (7.82)	589 (6.43)	8577 (93.57)
Smoker	16,831 (14.36)	1112 (6.61)	15,719 (93.39)
**Physical activity (Daily METs)**			
Inactive (24–36.5)	44,075 (33.89)	3673 (8.33)	40,402 (91.67)
Middle (36.6–44.9)	60,585 (46.59)	3.699 (6.11)	56,886 (93.89)
Active (≥45)	25,388 (19.52)	975 (3.84)	24,413 (96.16)
**BMI**			
Underweight	2558 (1.98)	155 (6.06)	2403 (93.94)
Normal	34,808 (26.90)	1933 (5.55)	32,875 (94.45)
Overweight	52,731 (40.76)	3291 (6.24)	49,440 (93.76)
Obese	39,279 (30.36)	2938 (7.48)	36,341 (92.52)
**Socioeconomic status**			
1st SES quintile (Poorest)	26,095 (20.06)	1813 (6.95)	24,282 (93.05)
2nd SES quintile	26,035 (20.01)	1777 (6.83)	24,258 (93.17)
3rd SES quintile	26,007 (19.99)	1766 (6.79)	24,241 (93.21)
4th SES quintile	25,940 (19.94)	1566 (6.04)	24,374 (93.96)
5th SES quintile (Richest)	26,001 (19.99)	1426 (5.48)	24,575 (94.52)
**Region of cohort (province)**			
Fars (FA)	22,767 (17.50)	1471 (6.46)	21,296 (93.54)
Kermanshah (KSH)	10,036 (7.72)	257 (2.56)	9779 (97.44)
Guilan (GU)	10,433 (8.02)	903 (8.66)	9530 (91.34)
East Azerbaijan (EA)	14,775 (11.36)	1072 (7.26)	13,703 (92.74)
Mazandaran (MA)	10,103 (7.77)	964 (9.54)	9139 (90.46)
Sistan and Balouchestan (SB)	8199 (6.30)	965 (11.77)	7234 (88.23)
Yazd (YA)	9723 (7.47)	577 (5.93)	9146 (94.07)
Kerman (KE)	9788 (7.52)	857 (8.76)	8931 (91.24)
Khuzestan (KH)	9139 (7.03)	148 (1.62)	8991 (98.38)
Chaharmahal and Bakhtiari (CB)	6730 (5.17)	450 (6.69)	6280 (93.31)
Hormozgan (HO)	3557 (2.73)	110 (3.09)	3447 (96.91)
West Azerbaijan (WA)	3660 (2.81)	157 (4.29)	3503 (95.71)
Ardabil (AR)	8214 (6.31)	372 (4.53)	7842 (95.47)
Razavi Khorasan (RK)	2954 (2.27)	45 (1.52)	2909 (98.48)

**Fig. 1:**
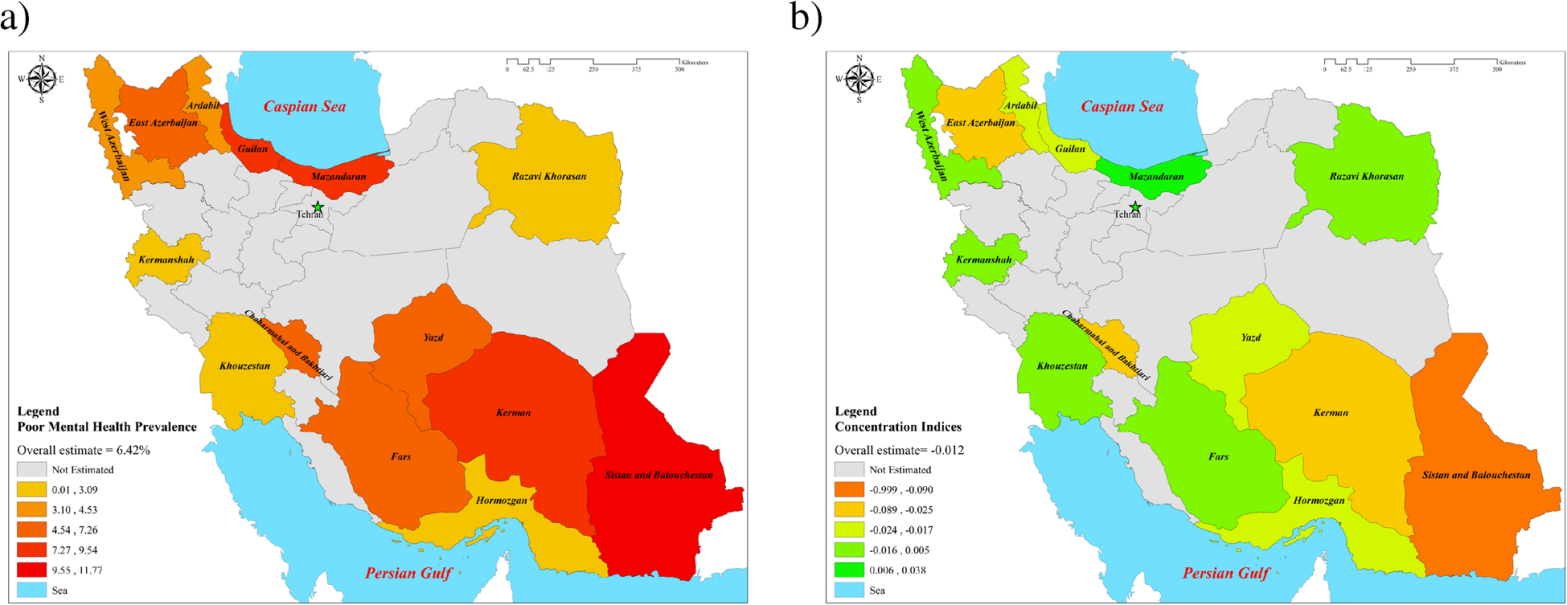
**a** Poor mental health prevalence by province of Iran. **b** The *C*_*k*_ of poor mental health by province of Iran

Age-adjusted proportion of poor mental health was 6.23% (95% Confidence Interval [CI]: 6.09, 6.36). The proportion of poor mental health among the females (8.14%) was higher compared to the males (4.29%). The cohort sites of Sistan and Balouchestan (11.77), and Razavi Khorasan (1.52) had higher and lower proportion of poor mental health among PERSIAN cohort sites (see Fig. 1a).

### Socioeconomic inequality in poor mental health

Table 2 and Fig. 1b show the Erreygers concentration indices (*C*_*k*_) for poor mental health for all separate sites of the PERSIAN cohort. The result shows that the overall E was − 0.012 (95% CI: − 0.0144 to − 0.0089), indicating the slightly higher concentration of poor mental health among less-advantaged people (*P* < 0.001). The values of *C*_*k*_ were not statistically significant for the Fars, Kermanshah, and Khuzestan sites. All the *C*_*k*_ values had negative signs, except for the Mazandaran site (*C*_*k*_ =0.0392, *P* < 0.001).

**Table 2:** The E for mental health disorders (*n* = 130,078)

	CI	95% Confidence Interval
**Overall estimate**	−0.012	− 0.015, − 0.009
**Gender**		
Female	− 0.002	− 0.007, 0.0025
Male	− 0.009	− 0.013, − 0.006
**Province**		
Fars (FA)	− 0.003	− 0.011, 0.003
Kermanshah (KSH)	− 0.004	− 0.012, 0.003
Guilan (GU)	−0.017	− 0.029, − 0.004
East Azerbaijan (EA)	− 0.025	− 0.035, − 0.016
Mazandaran (MA)	0.038	0.026, 0.050
Sistan and Baluchestan (SB)	− 0.090	− 0.107, − 0.074
Yazd (YA)	− 0.020	− 0.031, − 0.009
Kerman (KE)	− 0.028	− 0.041, − 0.016
Khuzestan (KH)	− 0.003	− 0.009, − 0.002
Chaharmahal and Bakhtiari (CB)	− 0.045	− 0.058, − 0.029
Hormozgan (HO)	− 0.018	− 0.029, − 0.006
West Azerbaijan (WA)	− 0.004	− 0.019, 0.010
Ardabil (AR)	− 0.020	− 0.031, − 0.010
Razavi Khorasan (RK)	0.005	− 0.006, 0.015

### Determinants of socioeconomic inequality in poor mental health

The results of the decomposition analysis are presented in Tables 3. The table presents elasticity, coefficient estimates, the *C*_*k*_, absolute contributions, and percentage contribution for each explanatory factor to poor mental health. A positive coefficient indicates that the participants with the explanatory variable were more likely to have poor mental health status and vice-versa.

**Table 3:** Decomposition of E of poor mental health (*n* = 130,078)

	Coefficient	Concentration index	Contribution	Contribution%	Summed
**Age groups**					12.703
35–40	Ref.	–	–	–	
40–45	.0894003	0.0524	0.0001	−1.2071	
45–50	.2150607	0.030	0.0002	−1.6621	
50–55	.3051117	−0.0048	−0.0000	0.3805	
55–60	.3049626	−0.0577	− 0.0005	4.5390	
60–65	.2760597	−0.0788	−0.0007	5.6091	
65 and older	.308196	−0.0635	−0.0006	5.0436	
**Gender**					19.927
Male	−.43360	0.1783	−0.0024	19.9270	
Female	Ref.	–	–	–	
**Marital status**					1.9963
Single	Ref.				
Married	−0.0611	0.0933	−0.0002	1.4656	
Divorced	0.0217	−0.0779	−0.0001	0.4353	
Widowed	0.0879	−0.005	−0.0000	0.1132	
Other	−0.0986	−0.0007	0.0000	−0.0178	
**Water-pipe use**					−1.1424
No	Ref.				
Yes	0.0623	0.0712	0.0001	−1.1424	
**Alcohol Use**					0.8492
No	Ref.				
Yes	−0.0487	0.0677	−0.0001	0.8492	
**Drug use**					0.3762
No	Ref.				
yes	0.1552	−0.0094	−0.0000	0.3762	
**Smoking status**					−0.1247
Non-smoker	Ref.				
Ex-smoker	0.21844	−0.0006	−0.0000	0.0336	
Smoker	0.12433	0.0049	0.0000	−0.1583	
**BMI**					−0.7472
Underweight	0.01042	−0.0225	−0.0000	0.0605	
Normal	Ref.				
Overweight	0.02785	0.0784	0.0001	−0.5632	
Obese	0.04223	0.0225	0.0000	−0.2445	
**Physical activity (Daily METs)**					−5.7784
Inactive (24–36.5)	Ref.				
Middle (36.6–44.9)	−0.1956	0.0703	−0.0004	3.5431	
Active (≥45)	−0.3385	−0.1068	0.0011	−9.3215	
**Socioeconomic status**					10.0531
1st SES quintile (Poorest)	Ref.				
2nd SES quintile	0.03825	−0.3191	−0.0004	3.1463	
3rd SES quintile	0.05155	0.0012	0.0000	−0.0154	
4th SES quintile	0.00051	0.3197	0.0000	−0.0421	
5th SES quintile (Richest)	−0.04223	0.6397	−0.0008	6.9643	
**Region of cohort (province)**					−11.0293
Fars (FA)	Ref.				
Kermanshah (KSH)	−0.3517	−0.0209	0.0002	−1.8938	
Guilan (GU)	0.19355	−0.0483	−0.0003	2.4108	
East Azerbaijan (EA)	0.1363	−0.0057	−0.0000	0.1992	
Mazandaran (MA)	0.3011	0.0461	0.0004	−3.5757	
Sistan and Balouchestan (SB)	0.3056	0.0082	0.0001	−0.6435	
Yazd (YA)	0.0654	0.0536	0.0001	−0.9046	
Kerman (KE)	0.1688	0.0847	0.0004	−3.6822	
Khuzestan (KH)	−0.6571	−0.0497	0.0010	−8.4243	
Chaharmahal and Bakhtiari (CB)	0.0956	0.0921	0.0003	−2.2701	
Hormozgan (HO)	−0.2641	−0.0056	0.0000	−0.3815	
West Azerbaijan (WA)	−0.1006	−0.0183	0.0001	−0.4738	
Ardabil (AR)	−0.1215	0.0551	−0.0002	1.7239	
Razavi Khorasan (RK)	−0.4975	0.0537	−0.0008	6.8863	
Total					27.083
Residual					72.917
The E			−0.012		100.0

Physical activity (Daily METs): A MET is equal to resting metabolic rate, the amount of oxygen consumed at rest that is about 3.5 ml 02/kg/min

Ref. indicated reference group in the regression estimation

The *C*_*k*_ was estimated for each explanatory factor of poor mental health. A negative (positive) sign shows that the explanatory variable has a pro-poor (pro-rich) distribution. Variables such as age categories of 50–55, 55–60, 60–65, and > 65, marital status categories of divorced, widows, and others, drug users, non-smokers, physically active and living in Cohort sites of Kermanshah, Guilan, East Azerbaijan, Khouzestan, Hormozgan, and West Azerbaijan had negative concentration indices, indicating that these predictors were concentrated among the pro-poor population. A negative (positive) absolute contribution of predictors means that socioeconomic inequality in poor mental health would, the value of *C*_*k*_, increase (decrease) if that predictor would be equally distributed across the SES distribution. All predictors included in the decomposition analysis explained 27.1% of overall inequality in poor mental health.

**Fig. 2:**
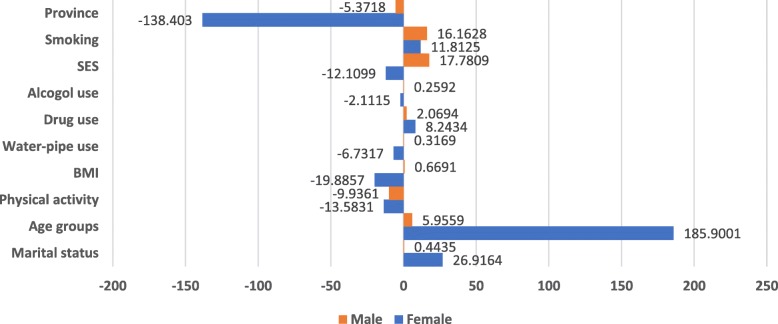
Percentage contribution to Erreygers CIs of poor mental health by gender

Gender explains the most of observed inequality in poor mental health . Figure 2 illustrates the results of decomposition analysis by gender. The contribution to *C*_*k*_ s of poor mental health of predictors such as, age groups, BMI and marital status, SES and province were varying between females and males. The contribution of SES to poor mental health inequality for female was negative (− 12.11%), while for the male was positive (17.78%). The contribution of province for both of the gender was negative (− 138.4% for female vs. -5.37% for male). The SES inequality in poor mental health for female and male are mainly explained by age (185.90%), and SES (17.78%), respectively.

## Discussion

The overall prevalence of poor mental health among Iranian adult was 6.2%. This is slightly lower than the result of previous studies conducted on general population in Iran [4, 17]. The observed inconsistency might be related to the different scale or approaches employed for mental health measurements. Our findings highlighted a substantial gender, and provincial heterogeneities in poor mental health distribution. The largest prevalence of poor mental health (11.7%) was observed in the cohort site of Sistan and Balouchestan province. This may be explained by a higher frequency of illicit drug use [18] and higher unemployment rate in this province [19]. Noorbala et al. [20] showed a decreased prevalence of mental disorders from 24.6% (1999) to 15.1% (2015) in Sistan and Balouchestan province [20]. They also found a higher prevalence in females (17.2%) as compared to males (13%). Consistent with literature, the prevalence of mental disorders in was more found to be more common in females and in older age group adults [20–23]. There was not a substantial difference in the poor mental health prevalence among 14 cohort sites.

Except for the Mazandaran site, the E for all sites had negative sign, suggesting a higher concentration of poor mental health in socioeconomically disadvantaged people. This is in line with the previous reports around the world [24–26]. However, the different methods employed for inequality measurement in various studies made the comparison of the concentration indices difficult.

The geographical inequalities in mental health might be explained by cultural differences across different regions of Iran. The geographical inequalities in health outcomes in Iran might be due to differences in unemployment rates, SES, and literacy levels for across different provinces [27]. Although, study by Movahedi et al. (2008) [28] demonstrated a decrease in the geographic inequality in some health indicators, the authors concluded that cross country differences in health remains an important public health problem in Iran.

The findings of this study demonstrated a more concentration of poor mental health among individuals with lower SES. Decomposing analysis demonstrated gender as the main contributor of observed inequality in the poor mental health prevalence. This is in line with the other inequality studies [29, 30] that suggested gender as a possible contributor to socioeconomic inequality in mental health. The contribution to E also showed that both sociodemographic (i.e. age, BMI, marital status, and SES) and geographical predictors (i.e. province) are varying between females and males. In a study by la Torre J et al. (2016) in Spain, mental health was mainly associated with SES among females [23]. Although, gender gaps in health and health-related factors is globally declining [31], it still exists in both developed and developing countries [32]. Females generally experience higher health-related outcomes including mental disorders, and healthcare services utilization [33]. SES of females are generally lower than males (see, for example, a positive value of the C for male in Table 3). The combination of poor mental health and lower SES of females as compared to males led to the significant contribution of gender factor to socioeconomic inequality in poor mental health in Iran. Demographic factors such as age and marital status also play an important role in the mental health inequalities in current study. Similar studies conducted in high- and middle-income countries have reported the same results [26, 34]. A study conducted by Morasae et al. (2012) in Tehran, demonstrated a contribution of 13.1% for age to socioeconomic inequality in mental health in Tehran, Iran [26]. In contrast with a study by Amroussia et al. [24], marital status had a small contribution to socioeconomic inequalities in poor mental health in Iran. Married people (compared to single, divorced and widowed) were found to have more contribution to socioeconomic inequality in poor mental health. This finding may be related to financial limitation that puts strains on marital relationship among couple. People from low socioeconomic groups usually experience more poor marital relationship, intimate partner violence, financial stress and pressure than those in high socioeconomic groups [35, 36].

There are a number of limitations need to be considered when interpreting the study results. First, the self-reported nature of drug use for mental health disorders might lead to recall bias. Second, because of the cross-sectional nature of the data, it was not possible to establish a causal correlation between explanatory variables and poor mental health outcome. Third, the estimated prevalence and the E may not be a representative sample of entire of Iran as data was not collected from some provinces in Iran.

## Conclusion

The findings of the study showed that poor mental health is nearly equitable distributed among Iranian adults, but with important variations by gender, SES, and geography. These results suggested that interventional programs in Iran should focus on should focus more on socioeconomically disadvantaged people as a whole, with particular attention to the needs of women and those living in more socially disadvantaged regions.

## Data Availability

The datasets analyzed during the current study are available from the corresponding author on reasonable request.

## Appendix

**Table 4:** The characteristics of the PERSIAN cohort sites included in the study

	Province	Population	Cohort site	Population	Cohort population	Main Ethnicities
1	Ardabil	1,270,420	Ardabil	529,374	8192	Azeri Turk
2	Chaharmahal and Bakhtiari	947,763	Sharekord	93,104	6664	Lor
3	East Azerbaijan	3,909,652	Khameneh	3056	14,978	Azari Turk
4	Fars	4,851,274	Kavar	31,711	2244	Fars (Persian), Turk
			Kharameh	18,477	10,662	Fars (Persian), Arab
			Fasa	110,825	10,113	Fars (Persian), Arab and Turk
5	Guilan	2,530,696	Some’e Sara	58,658	10,511	Gilaki
6	Hormozgan	1,776,415	Bandare Kong	19,213	3570	Arab
7	Kerman	3.164,718	Rafsanjan	161,909	9982	Fars (Persian)
8	Kermanshah	1,952,434	Ravansar	47,657	10,077	Kurd
9	Khouzestan	4,710,506	Hoveizeh	19,481	9156	Arab
10	Mazandaran	3,283,582	Sari	309,820	10,253	Tabari
11	Razavi Khorasan	6,434,501	Mashhad	3,001,184	2189	Fars (Persian)
			Sabzevar	243,700	784	Fars (Persian)
12	Sistan and Balouchestan	2,775,014	Zahedan	587,730	8318	Balouch
13	West Azerbaijan	3,265,219	Ghoushchi	2787	3662	Azeri Turk
14	Yazd	1,138,533	Shahedieh, Yazd	18,309	9901	Fars (Persian)

References: 1- Persian cohort sites, available from: http://persiancohort.com/cohortsites/, access: April 21, 2019. 2- Iran statistics center, available from: https://www.amar.org.ir, access: April 21, 2019

## Abbreviations

PERSIAN: Prospective epidemiological research in IrAN
SES: Socioeconomic status
BMI: Body mass index
C: Concentration index
E: Erreygers concentration index
