# Correction: Decomposing socioeconomic inequality in poor mental health among Iranian adult population: results from the PERSIAN cohort study

**DOI:** 10.1186/s12888-022-04194-6

**Published:** 2022-08-23

**Authors:** Farid Najafi, Yahya Pasdar, Behzad Karami Matin, Satar Rezaei, Ali Kazemi Karyani, Shahin Soltani, Moslem Soofi, Shahab Rezaeian, Alireza Zangeneh, Mehdi Moradinazar, Behrooz Hamzeh, Zahra Jorjoran Shushtari, Mansour sajjadipour, Saeid Eslami, Maryam khosrojerdi, Sahar Shabestari, Amir Houshang Mehrparvar, Zahra Kashi, Azim Nejatizadeh, Mohammadreza Naghipour, Shahrokh Sadeghi Boogar, Ali Fakhari, Bahman Cheraghian, Haydeh Heidari, Parviz Molavi, Mohammad Hajizadeh, Yahya Salimi

**Affiliations:** 1 Research Center for Environmental Determinants of Health, Health Institute, Kermanshah University of Medical Sciences, Kermanshah, Iran; 2 Nutritional Sciences Department, School of Public Health, Kermanshah University of Medical Sciences, Kermanshah, Iran; 3 Social Development and Health Promotion Research Center, Health Institute, Kermanshah University of Medical Sciences, Kermanshah, Iran; 4 Social Determinants of Health Research Center, University of Social Welfare and Rehabilitation Sciences, Tehran, Iran; 5 South Tehran Health Center, Tehran University of Medical Sciences, Tehran, Iran; 6 Pharmaceutical Research Center, Pharmaceutical Research Institute, Mashhad University of Medical Sciences, Mashhad, Iran; 7 Cellular and molecular research center, school of medicine, Sabzevar University of Medical Sciences, Sabzevar, Iran; 8 Health Promotion Research Center, Zahedan University of Medical Sciences, Zahedan, Iran; 9 Industrial Diseases Research Center, Shahid Sadoughi University of Medical Sciences, Yazd, Iran; 10 Diabetes Research Center, Mazandaran University of Medical Sciences, Sari, Iran; 11 Molecular Medicine Research Center, Hormozgan University of Medical Sciences, Bandar Abbas, Iran; 12 Gastrointestinal and Liver Disease Research Center, Guilan University of Medical Sciences, Rasht, Iran; 13 Gastroenterohepatology Research Center, Shiraz University of Medical Sciences, Shiraz, Iran; 14 Research Center of Psychiatry and Behavioral Sciences, Tabriz University of Medical Sciences, Tabriz, Iran; 15 Department of Biostatistics and Epidemiology, School of Public Health, Ahvaz Jundishapur University of Medical Sciences, Ahvaz, Iran; 16 Modeling in Health Research Center, Shahrekord University of Medical Sciences, Shahrekord, Iran; 17 Digestive Disease Research Center, Ardabil University of Medical Sciences, Ardabil, Iran; 18 School of Health Administration, Faculty of Health, Dalhousie University, Halifax, Canada; 19 Social Development and Health Promotion Research Center, Health Institute, Kermanshah University of Medical Sciences, Kermanshah, Iran

**Correction: BMC Psychiatry. 20, 229 (2020)**

**https://doi.org/10.1186/s12888-020-02596-y**

Following the publication of the original article [1], the authors would like to correct the code of the study in the ethics approval and consent to participate statement.

The incorrect code of the study is: IR.KUMS.REC.1397.187

The correct code of the study is: IR.KUMS.REC.1397.866

The original article [1] has been corrected.
